# The helminth product, ES-62 modulates dendritic cell responses by inducing the selective autophagolysosomal degradation of TLR-transducers, as exemplified by PKCδ

**DOI:** 10.1038/srep37276

**Published:** 2016-11-21

**Authors:** Russell J. Eason, Kara S. Bell, Fraser A. Marshall, David T. Rodgers, Miguel A. Pineda, Christina N. Steiger, Lamyaa Al-Riyami, William Harnett, Margaret M. Harnett

**Affiliations:** 1 Institute of Infection, Immunity and Inflammation, University of Glasgow, Glasgow G12 8TA, UK; 2 Strathclyde Institute of Pharmacy and Biomedical Sciences, University of Strathclyde, Glasgow G4 0RE, UK

## Abstract

We have previously shown that ES-62, a phosphorylcholine (PC)-containing glycoprotein secreted by the parasitic filarial nematode *Acanthocheilonema viteae* targets dendritic cell (DC) responses, specifically by suppressing TLR4 signalling to inhibit Th1/Th17-driven inflammation. We have now investigated the molecular mechanisms underpinning such immunomodulation and show here that ES-62-mediated downregulation of protein kinase C-δ (PKC-δ), a TLR4-associated signalling mediator required for full activation of LPS-driven pro-inflammatory responses, is associated with induction of a low level of autophagic flux, as evidenced by upregulation and trafficking of p62 and LC3 and their consequent autophagolysosomal degradation. By contrast, the classical TLR4 ligand LPS, strongly upregulates p62 and LC3 expression but under such canonical TLR4 signalling this upregulation appears to reflect a block in autophagic flux, with these elements predominantly degraded in a proteasomal manner. These data are consistent with autophagic flux acting to homeostatically suppress proinflammatory DC responses and indeed, blocking of PKC-δ degradation by the autophagolysosomal inhibitors, E64d plus pepstatin A, results in abrogation of the ES-62-mediated suppression of LPS-driven release of IL-6, IL-12p70 and TNF-α by DCs. Thus, by harnessing this homeostatic regulatory mechanism, ES-62 can protect against aberrant inflammation, either to promote parasite survival or serendipitously, exhibit therapeutic potential in inflammatory disease.

We have previously shown that ES-62, a phosphorylcholine (PC)-containing immunomodulatory glycoprotein secreted by the filarial nematode *Acanthocheilonema viteae*, is protective in mouse models of allergic^1^,^2^,^3^ and autoimmune^4^,^5^,^6^ disease. Such protection is associated with homeostatic resetting of the IL-23/IL-17/IL-22 inflammatory axis, which appears to become dysregulated in these inflammatory disorders: a key effector driving development of this type of inflammation is the dendritic cell (DC), which ES-62 targets by subverting TLR4 signalling^7^,^8^,^9^ to suppress aberrant DC-priming of Th17 cells and IL-17-producing γδ T cells^2^,^4^.

Although ES-62 requires TLR4 for its actions on DCs^7^,^8^, unlike the canonical TLR4 ligand LPS^10^, it does not signal for robust proinflammatory responses or downregulate TLR4 expression in these cells^7^,^8^,^11^. Rather, and more like the non-toxic species of LPS from *Rhodobacter sphaeroides*^12^,^13^ or *Bacteroides dorei*^14^, exposure to ES-62 dampens DC responses to challenge with LPS^7^,^8^,^11^. LPS internalises and trafficks TLR4 via a clathrin-dependent endosomal mechanism, initially to couple to the pro-inflammatory TRAM/TRIF signalling pathway and then ultimately, by promoting antigen (Ag)-presentation, to link innate and adaptive responses (reviewed in refs 10 and 11). Intriguingly, therefore, *R. sphaeroides* LPS and ES-62 are internalised and traffick TLR4 in a clathrin-independent manner (reviewed in ref. 11): indeed, and of relevance given the increasing evidence that caveolae/lipid rafts act as sensors to regulate signalling^15^, ES-62, which is internalised in a TLR4-dependent-manner in mast cells and macrophages^1^,^16^ induces the caveolae/lipid raft-dependent sequestration and autophagolysosomal degradation of TLR4-associated protein kinase C (PKC)-α in mast cells^1^,^17^.

The precise mechanism(s) underpinning differential LPS- and ES-62-mediated TLR4 signalling in DCs has yet to be fully resolved: however, our recent data indicating that ES-62, but not LPS, can drive the autophagolysosomal-mediated downregulation of MyD88 in DCs under hyper-inflammatory conditions^9^ suggests that ES-62 may generally act by harnessing homeostatic autophagy-dependent mechanisms to limit aberrant inflammation by degrading key TLR4 signal effectors. In support of this, whilst autophagy is a critical and complex cellular homeostatic mechanism with key roles in TLR-associated immunity, notably in killing of intracellular pathogens, antigen presentation and T cell polarisation, it also controls inflammation, acting to antagonise inflammasome signalling and to limit and resolve inflammation^18^. Therefore, we have now investigated whether ES-62 induces autophagolysosomal degradation of key effectors and known targets of the helminth product, such as the protein kinase C (PKC) isoforms (α, δ and ε) associated with the LPS/TLR4 cassette^19^,^20^, in an attempt to explain, how ES-62 subverts pro-inflammatory TLR4 signalling in DCs to reset Th cell polarisation.

We show that both LPS and ES-62 can upregulate components (p62 and LC3) of the autophagy machinery: however, whilst LPS-mediated pro-inflammatory responses are associated with (proteasomally-regulated) cytosolic p62 and PKC-δ signalling, and autophagic flux blockade, ES-62 acts to limit such cytosolic signalling by selectively inducing autophagolysosomal degradation of these elements. Moreover, blocking of autophagolysosomal degradation relieves ES-62-mediated suppression of LPS production of IL-6, IL-12p70 and TNFα by DCs, key mediators that contribute to pathogenic Th cell polarisation and inflammation in allergy and autoimmunity.

## Results

### ES-62 modulates DC function *in vitro* and *in vivo*

**Figure 1:**
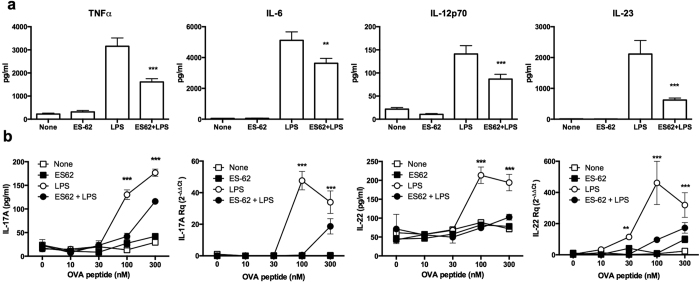
ES-62 inhibits LPS-stimulated DC responses. (**a**) Levels of TNFα, IL-6, IL-12p70, and IL-23 released by bmDCs derived from BALB/c mice incubated in medium alone (None) or containing ES-62 (2 μg/ml) for 18 h prior to culture in medium alone or containing LPS (1 μg/ml) for a further 18 h. Data are collated from 3 experiments and represent the means (of means of triplicate estimates from the independent experiments) ± SEM; **p < 0.01 and ***p < 0.001, relative to the LPS group. (**b)** BmDCs treated as described in **a**, were pulsed with OVA_323–339_ and co-cultured with OVA-specific KJ1.26^+^CD4^+^CD62L^+^ T cells for 72 h before measurement of IL-17A and IL-22 release by ELISA or determination of mRNA levels in the cells by qRT-PCR. Relative quantitation (Rq) of mRNA levels was defined by 2^−ΔΔCt values obtained relative to the house-keeping reference gene (GAPDH) and experimental control samples, namely the medium-only DCs (None; 0 nM OVA peptide). Data are the mean values ± SD, n = 3, from a single experiment where **p < 0.01 and ***p < 0.001 for ES-62+LPS-treated cells relative to the cells incubated with LPS alone. The ability of LPS-, but not ES-62-matured bmDCs from BALB/c mice to prime IL-17 release by KJ1.26^+^CD4^+^CD62L^+^ T cells was found in 3 additional independent experiments whilst LPS-, but not ES-62-matured bmDCs from C57BL/6 mice were shown to prime IL-17 and IL-22 release by OTII CD4^+^CD62L^+^ T cells in a further independent experiment (data not shown).

**Figure 2:**
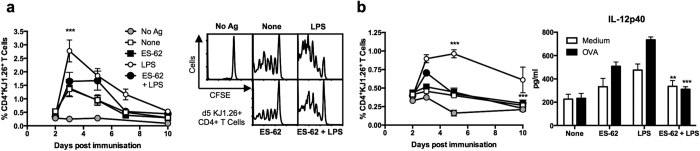
ES-62- and LPS-matured DCs exhibit differential responses *in vivo*. (**a**,**b**) BmDCs were cultured ± 2 μg/ml ES-62 and/or 10 μg/ml OVA_323-339_ and then ± LPS (1 μg/ml) prior to transfer (2.5 × 10^5^) to BALB/c mice that had previously received CFSE-labelled KJ1.26^+^CD4^+^ T cells (2.5 × 10^6^). Untreated bmDCs or those not loaded with OVA peptide provided “None” and “No Ag” controls, respectively. (**a)** Flow cytometric analysis of % of CD4^+^KJ1.26^+^ T cells in the DLN after DC inoculation presented as the mean values ± SEM of individual mice pooled from 2 independent experiments where ***p < 0.001 for LPS (n = 5) versus ES-62 + LPS (n = 6). Proliferation as indicated by CFSE staining is shown for a representative mouse from each group on day 5. (**b)** In a further independent experiment, the % of DLN CD4^+^KJ1.26^+^ T cells (means ± SEM, n = 3) after DC inoculation are shown along with IL-12p40 production by day 3 DLN cells, stimulated *ex vivo* with medium or OVA_323–339_ for 72 h. Data are mean values ± SEM, n = 3 individual mice, where **p < 0.01 and ***p < 0.001 for the relevant Medium or OVA-stimulation in the LPS versus ES-62 + LPS groups.

ES-62 exhibits the ability to modulate Th1-, Th17- and Th22-mediated responses, particularly when these phenotypes are dysregulated during development of pathogenesis in mouse models of rheumatoid arthritis (RA), systemic lupus erythematosus (SLE) and asthma^2^,^4^,^5^,^6^. For example, in the Collagen-Induced Arthritis (CIA) model of RA where Th1/Th17 responses are pathogenic, ES-62 targets DCs to suppress their priming of such aberrant Th and γδ T cells^4^. Consistent with this, ES-62 not only inhibits LPS-stimulated production of TNFα by DCs but also that of IL-6, IL-12p70 and IL-23, cytokines that promote development of IFNγ- and IL-17-producing T cells (Fig. 1a). Reflecting this, such ES-62-treated DCs exhibited reduced capacity to polarise Th17 or Th22 responses, both at the protein and mRNA level (Fig. 1b). Moreover, and mirroring the *in vivo* modulation of Ag-specific clonal expansion and Th1/Th17 cell polarisation by ES-62^21^, these effects are replicated *in vivo* as recipient mice receiving transfer of DCs exposed to ES-62+LPS show both reduced clonal expansion of OVA-specific Th cells (Fig. 2a,b) and production of proinflammatory cytokines associated with such effector responses upon restimulation of draining lymph node (DLN) cells *ex vivo* (Fig. 2b), relative to those receiving control LPS-matured DC (pre-treated with medium alone). Thus, although ES-62 and LPS both signal via TLR4 on DCs^7^,^8^, exposure of DCs to ES-62 alone does not stimulate the classical effector mechanisms (pro-inflammatory cytokine production and priming of Th1/Th17 proliferation and differentiation) of canonical TLR ligands, either *in vitro* or *in vivo*: these data therefore confirm and extend our evidence obtained to date (reviewed in refs 9 and 11) that ES-62 and LPS exploit differential TLR4 effector pathways.

### ES-62 selectively targets PKC-δ for downregulation in DCs

We have previously shown that the α, δ and ε isoforms of PKC regulate TLR4 signalling in mast cells and that ES-62 subverts such TLR4 signalling by downregulating expression of MyD88 and PKC-α and PKC-δ^1^,^17^,^22^: consistent with a similar mode of action in DCs, ES-62 acts homeostatically to limit expression of MyD88 which is upregulated in DCs under inflammatory conditions^9^. We therefore next investigated whether the α, δ and ε isoforms of PKC played key roles in LPS/TLR4-mediated cytokine responses in bmDCs and whether their differential signalling by ES-62 and LPS could provide a rationale for the observed distinct functional outcomes.

**Figure 3:**
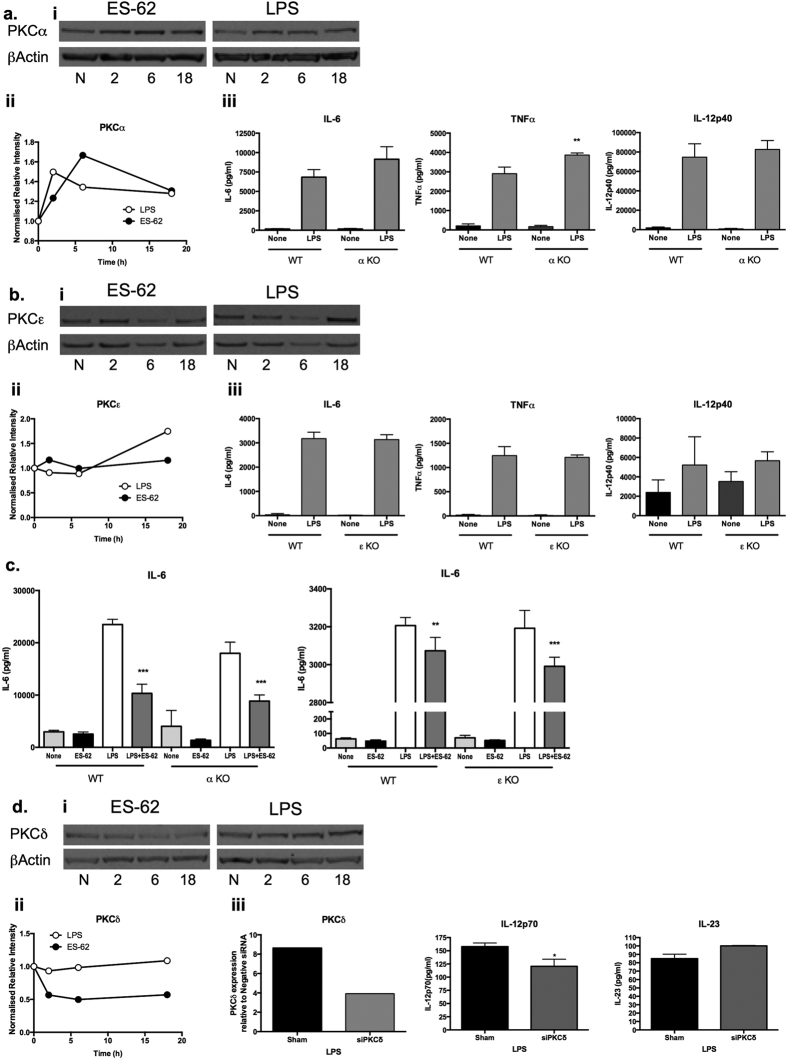
ES-62 selectively downregulates PKCδ expression in DCs. BmDCs were treated as described in Fig. 1 for up to 18 h with ES-62, LPS or incubated in medium alone (N, at 18 h time point) and analysed for PKC-α (**ai,ii)**, PKC-ε (**bi,ii**) or PKC-δ (**di,ii)** expression by Western blotting and quantitated relative to β-Actin and normalised to the 18 h medium control (N) from single experiments. Supporting the reproducibility of these findings, data normalised X-fold to the medium control from all the independent 18 h time-point Western blotting experiments performed in this study (other data not shown apart from relevant PKC-δ samples presented in Figs 4 and 7) are summarised here, expressed as mean values ± SEM where n = number of independent experiments, except in the case of PKC-α, where data are from 2 independent experiments and expressed as mean values. Thus for PKC-α: ES-62–2.17; LPS–1.28; PKC-ε: ES-62–0.77 ± 0.18, n = 4; LPS–1.56 ± 0.16, n = 4; PKC-δ: ES-62–0.56 ± 0.04, n = 12 p < 0.001; LPS–1.05 ± 0.13, n = 10. LPS-mediated cytokine release was measured by ELISA from DCs derived from matched WT or PKC-α (**aiii, c**) and PKC-ε (**biii, c**) mice. The data in **aiii** and **biii** are means ± SD, n = 3 from single experiments representative of at least 3 independent experiments whilst in **c**, the data are presented as means ± SD, n = 3 from single experiments. In diii, WT bmDCs were treated with control or PKC-δ-specific siRNA and then levels of PKC-δ expression determined by FACE assay and IL-12p70 or IL-23 measured by ELISA. In an independent experiment, treatment of DCs with siPKC-δ also suppressed LPS-stimulated IL-6 production by some 15% relative to the levels observed in the sham-treated control cells. Where indicated, *p < 0.05 **p < 0.01 and ***p < 0.001.

Firstly, analysis of PKC-α expression showed that exposure of DCs to either ES-62 or LPS resulted in some upregulation of this signalling element (Fig. 3ai,ii). The lack of any differential effect presumably reflects that PKC-α does not appear to be essential for LPS-stimulated cytokine secretion as indicated by experiments in bmDCs from PKC-α-deficient mice (Fig. 3aiii): rather, LPS-stimulated TNFα production appeared to be increased in such bmDCs. Similarly, although LPS, but not ES-62, induced an increase in PKC-ε expression (Fig. 3bi,ii), this isoform was also found to be dispensable for LPS-mediated cytokine responses (Fig. 3biii). Moreover, the ES-62-mediated inhibition of LPS-stimulated IL-6 responses was found to be intact in bmDCs from either PKC-α or -ε KO mice (Fig. 3c).

Interestingly therefore, given that PKC-δ plays key roles in DC development and motility, IL-12p40/p70 production, MHC II-Ag presentation and polarisation of Th1 responses^23^,^24^,^25^,^26^, whereas ES-62 reproducibly decreased (0.56 ± 0.04 of control value at 18 h, n = 12 independent experiments p < 0.001) expression of this isoform (Fig. 3di,ii), LPS had little effect (1.05 ± 0.13 of control value at 18 h, n = 10 independent experiments). As PKC-δ plays a key role in development of DCs from haemopoietic stem cells^26^, rather than derive bmDCs from PKC-δ-deficient mice, we tested the effect of PKC-δ-specific siRNA on LPS-mediated DC responses: this showed that, as with ES-62, partial knockdown of PKC-δ expression by siPKC-δ resulted in significant (~25%) inhibition of LPS-mediated IL-12p70 release but perhaps surprisingly, had no effect on the production of another IL-12 family cytokine, IL-23 (Fig. 3diii).

### ES-62 induces autophagolysosomal degradation of PKC-δ in DCs

**Figure 4:**
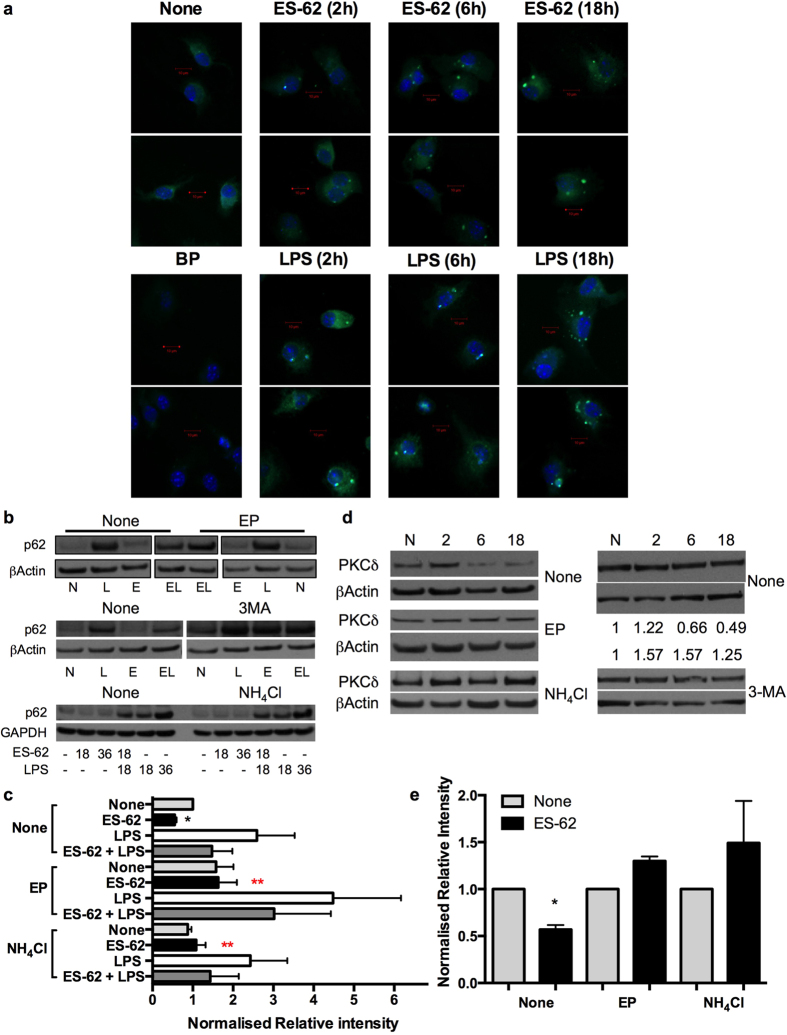
ES-62 downregulates PKC-δ via autophagolysosomal degradation. (**a)** Following incubation in medium (None) ± LPS or ES-62 for the indicated time DCs were stained for p62 (green; specificity demonstrated by p62 epitope blocking peptide [BP]) and cell nuclei (DAPI; Blue). Scale bars are shown in red (10 μm; magnification x63; scan zoom 1; digital zoom 2). (**b)** DCs pre-treated with autophagolysosomal inhibitors E64d plus pepstatin A (EP), 3MA or not (None) were incubated in medium ± ES-62 for 18 h and then incubated in medium ± LPS for a further 18 h, annotated as medium alone (N), ES-62 (E), LPS (L) or ES-62+LPS (EL). Alternatively, DCs treated with NH_4_Cl or not (None) were cultured with these modulators for the times (hours) indicated. p62 expression was determined by Western blot analysis. (**c**) The effect of EP or NH_4_Cl on ES-62- and/or LPS-modulation of p62 expression at 36 h, as determined by Western Blotting relative to the appropriate medium control (None/None). Data represent mean values ± SEM, collated from a number of independent experiments (n values) where *p < 0.05 for ES-62 relative to the None/None and None/LPS (all n = 6) but not None/ES-62+LPS (n = 4) groups and red **p < 0.05 for ES-62 versus ES-62 + EP (n = 4) and ES-62+NH_4_Cl (n = 3). All data presented are from at least 3 independent experiments, apart from those in the NH_4_Cl/ES-62 + LPS group, where data are from 2 independent experiments and presented as mean ± range. (**d**) DCs were incubated in medium alone (N; 18 h) or containing ES-62 for 2, 6 or 18 h as indicated in the presence and absence of the specified inhibitors and PKC-δ expression determined by Western Blot analysis. Quantitative analysis of the effect of 3MA on PKC-δ expression (fold medium control) is shown (**d;** right hand panels). (**e)** Data for the effects of EP and NH_4_Cl on ES-62-mediated downregulation of PKC-δ expression at 18 h, normalised to the relevant medium control (None) are collated from 7 independent experiments for the None (no inhibitor) cohorts and from 3 independent experiments for each of the EP and NH_4_Cl cohorts and presented as means ± SEM, where *p < 0.05.

Currently, the role(s) of autophagy in TLR-mediated innate and adaptive immunity remains to be fully delineated, as the complexity of the system has resulted in data supporting both inflammation-promoting and -resolving roles for this apparently critical cellular homeostatic mechanism^18^. In any case, stimulation of DCs with either ES-62 or LPS induces cellular trafficking of the key autophagy element, p62 (Fig. 4a) to vesicular compartments, as evidenced by punctate staining in confocal microscopy. Interestingly, therefore, although LPS upregulates p62 (Fig. 4b,c), this increased expression (2.59 ± 0.94 fold at 18 h, n = 6 independent experiments) is not significantly enhanced by treatment of the cells with a variety of inhibitors (E64d plus pepstatin A, NH_4_Cl or 3-methyladenine [3MA]) that target different aspects of the autophagolysosomal pathway^27^,^28^, suggesting a potential block in autophagic flux in LPS treated cells.

By contrast, ES-62 induces downregulation of p62 (0.55 ± 0.05 fold at 18 h relative to the None group, n = 6 independent experiments) to levels that are significantly different to those observed in either control (None) or LPS-, but not ES-62 plus LPS-treated cells (Fig. 4b,c), suggesting that exposure to ES-62 can also antagonise the LPS-mediated upregulation of p62: however, this modulation is somewhat lost under conditions suppressing autophagic flux where, apart from the prevention of ES-62-mediated downregulation of p62, there are no significant differences in expression of this transducer amongst the treatment groups (Fig. 4b,c).

Collectively, these data suggest that ES-62, but not LPS, is inducing autophagic flux as evidenced by its apparent autophagolysosomal degradation of components of the autophagy machinery, like p62. They also suggest that ES-62 may exploit this homeostatic mechanism to selectively degrade certain key signalling elements, like PKC-δ^19^,^20^,^29^ to subvert classical TLR4 signalling: consistent with this, analysis of the effect of the autophagolysosomal inhibitors on PKC-δ expression reveals that treatment with these prevents the degradation resulting from exposure to ES-62 (Fig. 4d,e).

**Figure 5:**
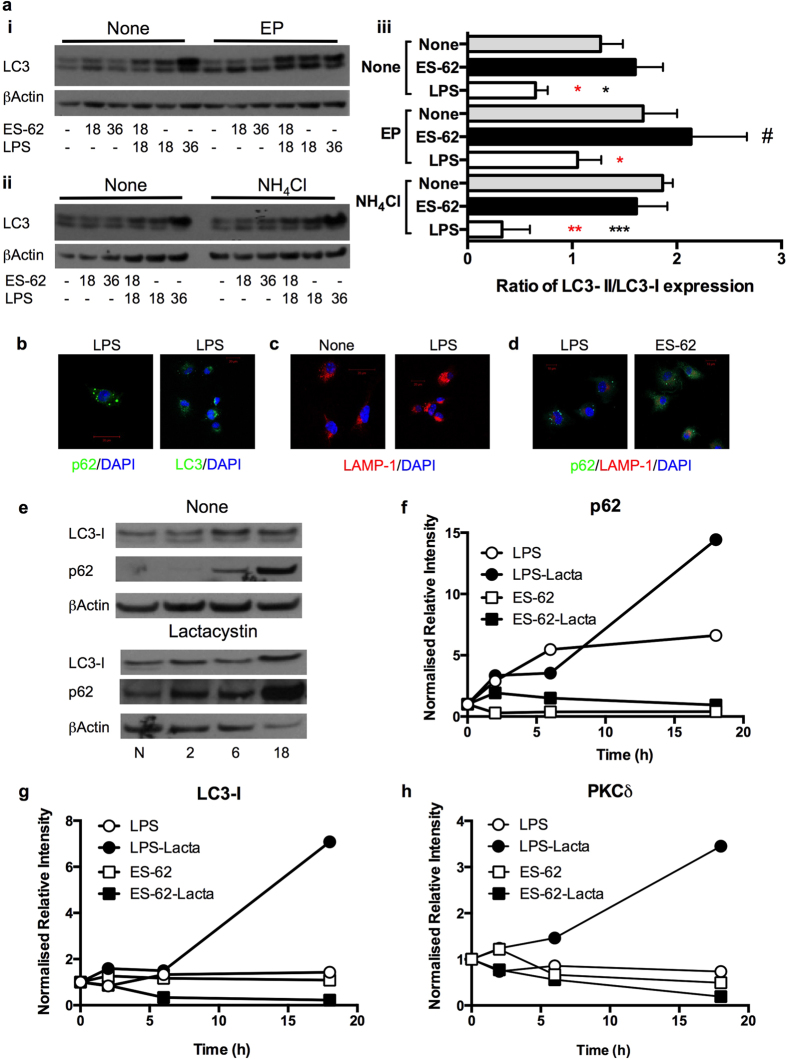
ES-62 stimulates autophagic flux. (**ai–iii**) DCs were incubated in medium ± ES-62 and/or LPS for the indicated time in the presence and absence of EP **(i)** or NH_4_Cl **(ii)** and LC3-I (upper band) and LC3-II (lower band) expression determined by Western blot analysis. Via quantitative analysis, the levels of LC3-I and LC3-II were normalised to the β-actin loading control and then the ratio of LC3-II/LC3-I (LC3-II/β-actin: LC3-I/β-actin) determined and statistical analysis **(iii)** of the effects of the inhibitors on the responses to ES-62 or LPS at 18 h undertaken. Data are presented as means ± SEM and were collated from the following number (n) of independent experiments: None (no inhibitor) cohorts, n = 6; E64d+pepstatin A cohorts, n = 3; NH_4_Cl cohorts, n = 4. *p < 0.05 and ***p < 0.001 are for the relevant LPS versus None groups, red */** represent comparisons between relevant LPS and ES-62 groups and ^#^p < 0.05 for ES-62-EP versus the None/None group. No significant differences were detected amongst the LPS groups. (**b–d**) Following incubation in medium (None) ± LPS or ES-62 for 18 h as indicated, DCs were washed, fixed and permeabilised and stained for: (**b**) p62 or LC3 (green) expression (magnification x63, scale bars, 20 μm); (**c**) LAMP-1 (red) expression; (**d**) p62 (green) and LAMP-1 (red) expression, with cell nuclei counterstained with DAPI (blue; magnification x63; scale bars, 10 μm). Data in (**b**–**d**) are from independent experiments, and distinct from the experiment presented in Fig. 4. (**e)** DCs were incubated in medium (N for 18 h) or with LPS in the presence or absence (None) of lactacystin for 2, 6 or 18 h as indicated and p62 and LC3-I expression determined by Western Blot analysis. Relatively under-exposed films are shown to allow for visual demonstration of the strong increase in their expression in the presence of lactacystin. Quantitative analysis of the effect of lactacystin on the ES-62- and/or LPS-modulation of p62 (**f**), LC3-I (**g**) and PKC-δ (**h**) expression at the indicated times relative to the appropriate medium control is presented and data are from single experiments.

Further support for ES-62, but not LPS, inducing autophagic flux is provided by Western Blot analysis (Fig. 5ai,ii) of expression of LC3-I and LC3-II as the ratio of LC3-II/LC3-I expression (Fig. 5aiii) reflects the dynamic phosphatidylethanolamine conjugation and autophagy vesicle association of LC3-II generated from the cytosolic LC3-I form^27^. This reveals that whilst LPS can stimulate an increase in LC3-I and to a lesser extent, LC3-II expression (relative to β-actin), neither molecule is substantially affected by treatment with either E64d plus pepstatin A or NH_4_Cl. Rather, LPS-stimulation for 36 h appears to result in a decrease in the LC3-II/LC3-I ratio relative to that observed in untreated (None) cells (Fig. 5ai: None/None, 1.306, None/LPS, 0.545; Fig. 5aii: None/None, 2.027, None/LPS, 0.144, where values shown are the LC3-II/LC3-I ratios). This decrease is maintained even in the presence of the autophagolysosomal inhibitors (Fig. 5ai: EP/None, 2.044, EP/LPS, 0.600; Fig. 5aii: NH_4_Cl/None, 1.620, NH_4_Cl/LPS, 0.102).

By contrast, treatment with ES-62 for 36 h increases the LC3-II/LC3-I ratio in the absence and presence of the inhibitors (Fig. 5ai: None/ES-62, 1.614, EP/ES-62, 2.473; Fig. 5aii: None/ES-62, 2.505, NH_4_Cl/ES-62, 1.795) relative to the appropriate control group. Moreover, pretreatment with ES-62 can reduce the LPS block in the presence and/or absence of E64d plus pepstatin A or NH_4_Cl (Fig. 5ai: None/ES-62 + LPS, 0.590, EP/ES-62 + LPS, 0.977; Fig. 5aii: None/ES-62 + LPS, 1.072, NH_4_Cl/ES-62 + LPS, 1.102). Analysis of several independent experiments shows that the effects of ES-62 at 18 h do not achieve statistical significance relative to the relevant control group: nevertheless, the LC3-II/LC3-I ratio levels pertaining in the EP/ES-62 group are significantly higher (p < 0.05) than in the None/None cohort (Fig. 5aiii), supporting the proposal that ES-62 induces dynamic autophagic flux^27^. Moreover, this analysis does confirm the significant suppression of autophagic flux elicited by LPS relative to untreated (None) or ES-62-exposed cells at 18 h, even in the presence of the inhibitors of autophagic flux.

Furthermore, and consistent with TLR4 signalling inducing a block in autophagic flux in DCs, LPS induces a distinct pattern of p62 and LC3 staining in bmDCs (Fig. 5b) and a strong accumulation and disperse staining of the lysosomal marker, LAMP-1 (Fig. 5c) that is also distinct from the punctate distribution of p62 in such cells (Fig. 5b,d): by contrast, there is evidence of a low level of p62 and LAMP-1 co-localisation in ES-62-treated cells (Fig. 5d). Intriguingly, therefore, treatment with the proteasomal inhibitor, lactacystin appears to strongly enhance the levels of p62, LC3-I and PKCδ in LPS- but not ES-62-treated cells (Fig. 5e–h), suggesting that under steady-state conditions, TLR4 signalling might normally target proteasomal degradation of p62/LC3 to prevent autophagy homeostatically limiting (PKCδ-mediated) DC cytokine production, and hence allow initiation of pro-inflammatory responses.

### Blocking of autophagolysomal degradation does not convert ES-62 to a classical TLR4 ligand but inhibition of the proteasome limits LPS responses in DCs

**Figure 6:**
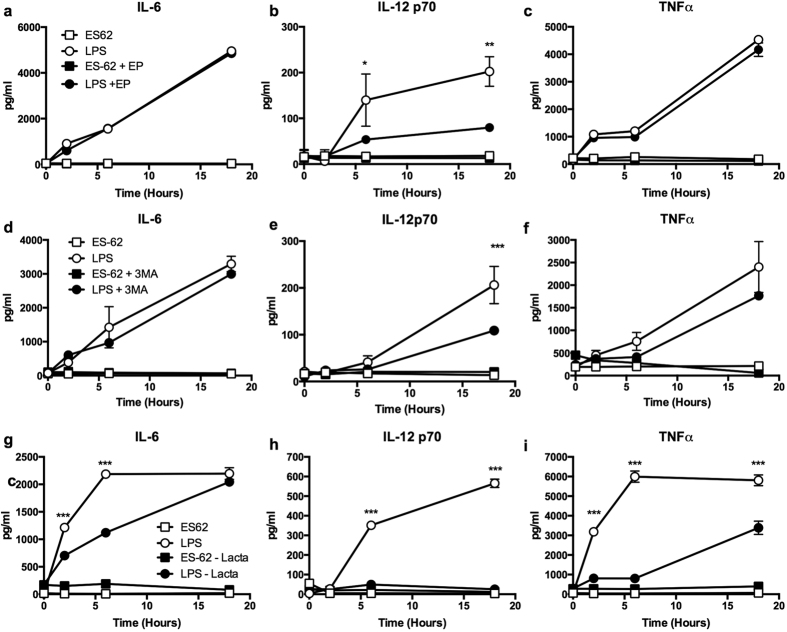
Autophagolysomal inhibitors do not convert ES-62 to a classical TLR4 ligand. DCs were treated with ES-62 or LPS for the indicated times in the absence and presence of E64d + pepstatin A (EP, **a–c**), 3MA (**d–f**) or lactacystin (Lacta, **g–i**) and the levels of IL-6 (**a,d,g**), IL-12p70 (**b,e,h**) and TNFα (**c,f,i**) determined. Data are derived from single experiments (means ± SD, n = 3), where *p < 0.05, **p < 0.01 and ***p < 0.001.

The differential proteasomal and autophagolysomal targeting of LPS and ES-62 responses suggested that blocking of their respective degradation machinery might allow conversion of ES-62-TLR4 signalling to a classical LPS phenotype, or *vice versa*. However, treatment with E64d plus pepstatin A (Fig. 6a–c) or 3MA (Fig. 6d–f) did not result in ES-62 inducing LPS-like cytokine production and indeed, these inhibitors generally had little apparent effect on either ES-62 or LPS responses, although LPS-stimulated IL-12p70 production was reduced (Fig. 6b,e). By contrast, whilst treatment with lactacystin only marginally modulated ES-62 responses, it inhibited LPS-stimulated IL-6, IL-12p70 and TNFα production by the DCs (Fig. 6g–i).

### Blocking of autophagolysomal degradation differentially modulates ES-62-mediated inhibition of LPS-stimulated cytokine production in DCs

**Figure 7:**
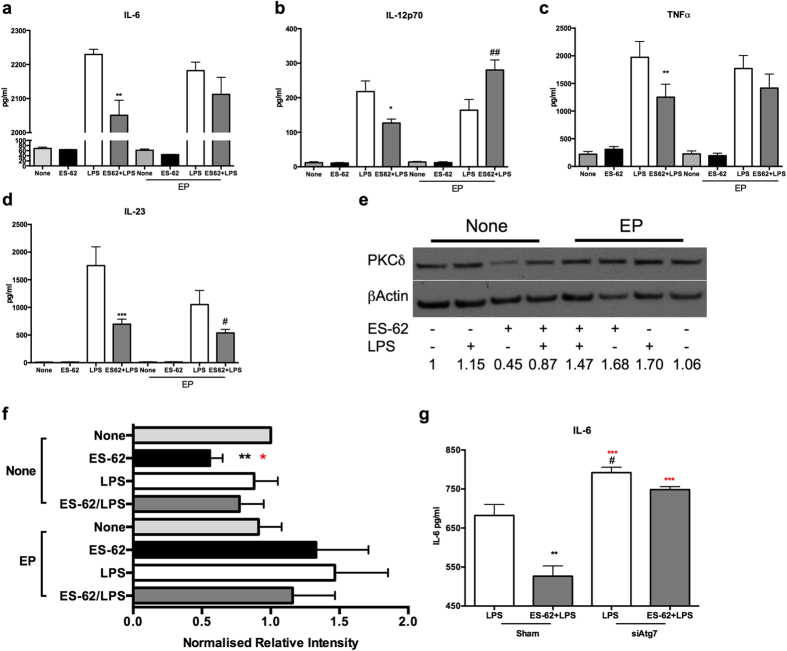
Blockade of autophagic flux prevents ES-62-mediated inhibition of LPS-stimulated IL-6, TNFα and IL-12p70 but not IL-23 production by DCs. DCs were incubated in medium (None) ± ES-62 for 18 h and then with medium ± LPS for a further 18 h in the presence and absence of E64d plus pepstatin A (EP) and the levels of IL-6 (**a**), IL-12p70 (**b**), TNFα (**c**), and IL-23 (**d**) released, shown. Data in (**a**) are from a single experiment (means ± SD, n = 3) whereas in b-d, data are the means ± SEM of means collated from three independent experiments, where *p < 0.05, **p < 0.01 and ***p < 0.001 relative to the LPS group and ^#^p < 0.05 and ^##^p < 0.01 relative to the LPS-EP group. DCs were incubated in medium ± ES-62 for 18 h and then medium ± LPS for a further 18 h in the presence and absence of EP and PKC-δ expression determined by Western Blot analysis (**e**) with quantitation of PKC-δ expression in this experiment relative to None/None control shown on the figure. Analysis was of data collated from 5 independent experiments for the None (no inhibitor) cohorts and 3 independent experiments for the EP cohorts (**f**) with data presented as means ± SEM where **p < 0.01 for ES-62 versus None and red*p < 0.05 for ES-62 versus LPS. In g, WT bmDCs were treated with control or ATG7-specific siRNA and then cultured in medium ± ES-62 prior to stimulation with LPS as indicated and then the levels of IL-6 measured by ELISA. Data are presented as means ± SD, n = 3 from a single experiment where **p < 0.01 is relative to the LPS-Sham, red***p < 0.001 relative to ES-62+LPS-Sham and, ^#^p < 0.05 relative LPS-sham.

Although blocking of autophagolysomal degradation did not convert ES-62 to a classical TLR4 ligand, the key role of PKC-δ in IL-12p70 production in DCs^25^ confirmed here (Fig. 3d) suggested that treatment with E64d plus pepstatin A might prevent the ES-62-mediated inhibition of LPS-induced cytokine production by DCs. This indeed turned out to be the case for IL-6, IL-12p70 and TNF-α (Fig. 7a–c) but not IL-23 (Fig. 7d) production: however, the lack of effect on IL-23 release was consistent with that observed in the cells treated with the PKC-δ-specific siRNA (Fig. 3diii). Moreover, analysis of PKC-δ levels revealed that whilst ES-62 reduced the levels of PKC-δ expression in DCs under conditions of steady-state and somewhat, under LPS stimulation, this downregulation was not observed in the presence of the autophagolysomal inhibitors (Fig. 7e,f).

Finally, further support for autophagolyosmal-mediated degradation of TLR-associated transducers like PKC-δ playing a role in the ES-62-mediated dampening of inflammatory responses was provided by the finding that whilst treatment with siPKC-δ could mimic the effect of ES-62 in inhibiting LPS-stimulated IL-12p70 and IL-6 production (Fig. 3diii and legend), exposure to siATG7 to block autophagic flux by disrupting the PE-conjugation of LC3-I, not only resulted in the enhancement of LPS responses but abrogated their inhibition by ES-62 (Fig. 7g).

## Discussion

ES-62 is a PC-containing immunomodulatory glycoprotein that exhibits protective effects in models of allergic and autoimmune disease^9^,^11^ by homeostatically resetting the effector: regulatory B cell balance and consequently modulating the IL-23/IL-17/IL-22 inflammatory axis^2^,^3^,^4^,^5^,^6^,^30^, dysregulation of which has been implicated in the pathogenesis of chronic inflammatory disorders^31^. Although the precise cellular networks modulated by the worm product appear to differ depending on the inflammatory context, ES-62 likely affords protection by acting to subvert TLR signalling via partial downregulation of MyD88 expression in effector cells of both innate and adaptive immunity^3^,^4^,^6^,^9^,^17^. The effect of targeting this key integrator of inflammatory signalling is exemplified by the ability of ES-62 to suppress both the priming and maintenance of pathogenic Th17 and IL-17-producing γδ T cells in CIA by targeting MyD88 in both DCs and Th17 cells^4^,^9^. Moreover, this data explains at least in part how, despite signalling via TLR4/MyD88, ES-62 does not mimic canonical TLR4 ligands in driving proinflammatory responses and indeed can inhibit cellular responses to subsequent TLR-2, -4, -9, but not TLR-3, ligands^4^,^7^,^8^,^22^,^32^. However, ES-62 is not immunosuppressive *per se* allowing animals and humans, harbouring PC-containing molecules like ES-62 in their bloodstream, to mount protective responses against infection whilst dampening those resulting from aberrant inflammation (reviewed in refs 9 and 33).

This lack of generalised immunosuppression has presumably arisen over the millennia of host-parasite evolution, generating a “safe” mechanism of immunomodulation tailored to specific aberrant inflammatory contexts and suggesting that the partial downregulation of MyD88 selectively targets certain regulatory nodes of the TLR4 (and other TLR) signalling cascades amplified in allergy and autoimmunity. In investigating this hypothesis here, we focused on the role of PKC-α, -δ and -ε isoforms not only because of their well-established roles in mediating proinflammatory TLR signalling in innate cells^19^,^20^,^34^ but also because these have been reported to directly associate with TLR-MyD88-Mal complexes^29^,^35^,^36^,^37^. Intriguingly, we have previously found ES-62 to differentially regulate the expression of these PKCs depending on the cell lineage, or even functional phenotype and/or receptor, targeted^1^,^11^,^17^,^22^. Given the important roles reported for PKC-α in DCs^37^ and our findings that ES-62 downregulates its expression in mast cells and B and T cells^1^,^11^,^17^, we were initially rather surprised that ES-62 did not exhibit differential effects to LPS on expression of this isoform: however, our analysis of DCs derived from PKC-α-deficient mice showed this isoform to be dispensable for full TLR4 signalling, at least in terms of IL-6, TNF-α and IL-12p40 production. Likewise, consistent with reports that it associates with MyD88^36^ and mediates LPS-stimulated IL-12-dependent responses in human monocyte-derived DCs^38^ we find that PKC-ε is upregulated by LPS, but not ES-62. However, this PKC isoform also appears to be non-essential for LPS-induced cytokine responses in murine bm-derived DCs, perhaps indicating differential roles in DC subtypes or alternatively, redundancy in PKC isoforms.

By contrast, and in keeping with PKC-δ playing key roles in DC development and motility, IL-12p40/p70 production, MHC II-Ag presentation and polarisation of Th1 responses^23^,^24^,^25^,^26^, we found ES-62, but not LPS, to downregulate expression of PKC-δ, typically to about 50% of the levels found under steady-state conditions. The functional relevance of this downregulation was indicated by the siRNA-mediated partial knockdown of this isoform also resulting in inhibition of LPS-induced IL-6, IL-12p70, but not IL-23, production. Interestingly, therefore, downregulation of PKC-δ has been shown to similarly suppress TLR-mediated cytokine production in monocytes by inhibiting NF-κB activation^29^,^39^, perhaps reflecting reports that PKC-δ can phosphorylate IκB resulting in its subsequent ubiquitination and degradation by the 26S proteasome, thereby allowing nuclear translocation of NF-κB and its binding to κB elements^40^,^41^.

Interestingly, given that we have found MyD88 expression in DCs to be homeostatically regulated by ES-62 in an autophagolysosomal manner, as indicated by the blockade of its downregulation by the inhibitors, E64d plus pepstatin A^9^, we show that the selective degradation of PKC-δ by ES-62 is associated with low level, dynamic autophagic flux resulting in its autophagolysomal degradation. This is indicated by immunofluorescence analysis of p62 and LAMP-1 trafficking and Western blot-detected increases in p62, LC3-II/I ratio and PKCδ expression in response to ES-62 in the presence of a variety of inhibitors of autophagic flux^27^,^42^. By contrast, although LPS induces upregulation and redistribution of p62 and LC3, these are not further increased by autophagolysosomal inhibitors and consistent with this, LPS does not direct p62 and LC3 or LAMP-1 co-localisation: collectively, these effects of LPS are more consistent with blockage of autophagic flux^27^,^42^,^43^. Indeed, LPS-mediated upregulation of p62 and LC3 elements appears to be greatly enhanced by the proteasome inhibitor, lactacystin suggesting that this TLR4 ligand also acts to sequester p62 and LC3 from the autophagy machinery, data perhaps in keeping with proposals that although TLR-signalling promotes autophagy to effect microbial elimination, induction of autophagic flux may be a key player in the complex homeostatic regulatory networks acting to limit inflammation^18^.

By inducing low-level autophagic flux, ES-62 may be harnessing this homeostatic network to dynamically prevent hyperinflammatory responses, a proposal supported by the finding that treatment with E64d plus pepstatin A prevents the ES-62-mediated downregulation of IL-6, TNFα and IL-12p70. That this is harnessing of a homeostatic mechanism to limit inflammation is perhaps further supported by our findings that use of the autophagolysosomal inhibitors does not convert ES-62 into a conventional TLR4 ligand. By contrast, and reflecting independent studies in macrophages^44^, proteasomal blockade appears to delay and diminish LPS-stimulated cytokine production, perhaps by permitting some autophagic flux due to the resulting increased levels of p62. Nevertheless, the strong LPS-induced cytosolic p62 signalling observed is reminiscent of that reported in keratinocytes^45^ where although TLR-signalling promotes p62-dependent autophagy to negatively regulate inflammation, p62, in keeping with its ability to activate NF-κB in autophagy-defective tumour cells^46^,^47^, also promotes NF-κB activation and consequent inflammatory responses.

Intriguingly, given the ability of LPS, but not ES-62, to upregulate PKC-ε, p62 has been proposed to act as an atypical PKC scaffold to generate a PKC-ε/p62/Traf6 complex that is critical for the activation of NF-κB in response to IL-1, TNFα and RANKL^48^,^49^ leading to the suggestion that upregulation of cytosolic p62 may, by promoting NF-κB activation, prime TLR-driven inflammatory responses^45^. Thus, given our preliminary data that ES-62 also downregulates Traf6 (Eason, Harnett & Harnett, unpublished), the lack of strong cytosolic p62 signalling induced by this non-canonical TLR4 ligand may go some way to explaining why blockage of autophagic flux does not convert ES-62 to a classical TLR4 ligand. Indeed, treatment with lactacystin appears to be able to prevent the early (<6 h) ES-62-mediated downregulation of p62, suggesting that ES-62 can target both the NF-κB (via the proteasome) and the autophagy-promoting capabilities of p62. This capacity to potentially induce both proteasomal and autophagolysosomal degradation of p62 is reminiscent of the ability of ES-62 to exploit both proteolytic compartments to downregulate PKCα in mucosal-like bm-derived and peritoneal-derived mast cells but not connective tissue mast cells (autophagoloysomal only)^17^. Collectively, therefore our data suggest that by inducing autophagolysomal and proteasomal degradation of key signalling elements, ES-62 can homeostatically rebalance aberrant DC-primed Th1 and Th17 responses.

In conclusion, millenia of host-pathogen co-evolution has generated immunomodulators, like ES-62, that act to dampen down host inflammation and limit tissue pathology^11^,^50^ thereby promoting both parasitic worm and host survival. This, in conjunction with the clear inverse relationship between the incidence of parasitic helminths and the prevalence of chronic allergic and autoimmune inflammatory disorders globally, has, via the Hygiene Hypothesis, generated increasing interest in exploiting such parasites and/or their immunomodulators as novel and theoretically safer therapies in chronic human inflammatory diseases^11^,^51^,^52^,^53^. Thus, discovering how immunomodulators like ES-62 exploit homeostatic regulatory mechanisms, not only informs our fundamental understanding of inflammation and its resolution, but can also identify potential novel and safer nodes of intervention in chronic inflammatory disease. Importantly, as ES-62 harnesses autophagic flux to selectively degrade TLR signalling elements, transducers that may differ, depending on cell type and/or TLR ligand, this provides the potential to homeostatically normalise inflammatory responses irrespective of their phenotype.

## Materials and Methods

### Animals and ES-62

All animals were maintained under standard *ad libitum* conditions at the Universities of Glasgow and Strathclyde SPF Biological Services Facilities and all experimental procedures were approved by, and performed in accordance with, the Animal Welfare and Ethical Review Body at the University of Glasgow, the Ethical Review Board of the University of Strathclyde and UK Home Office Regulations and Licenses PPL 60/3046, 60/2795 and 60/4300. Male, 6–8 week old, wild type BALB/c and C57BL/6 mice purchased from Harlan Olac (Bicester, U.K.) were used to generate bone marrow (bm)-derived dendritic cells (DCs) and DO.11.10.BALB/c mice and OT-II.C57BL/6 mice (both bred in-house) were used as sources of ovalbumin (OVA_323–339_)-specific transgenic (tg) TCR CD4^+^ T cells^4^,^21^,^54^,^55^. Bm-DCs derived from BALB/c mice and OVA_323–339_-specific tg TCR CD4^+^ T cells from DO.11.10.BALB/c mice were used as donor cells for adoptive transfer to recipient BALB/c mice. For PKC isoform^−/−^ studies, PKC-α (C57BL/6-Sv129 background) and –ε (C57BL/6Jax background) deficient mice in conjunction with their age and sex matched wild-type controls were used to derive bmDCs. PKC-α^−/−^ and PKC-ε^−/−^ mice were generated as previously described^22^. Bones from each set of PKC-isoform-deficient mice and their relevant wild-type genetic background controls were shipped to the University of Strathclyde from the donor laboratory group’s animal unit. Endotoxin-free ES-62 was prepared and purified from culture medium following maintenance of adult *Acanthocheilonema viteae* as described previously^56^.

### Analysis of DC function

DCs were derived from femur bone marrow cells from either BALB/c or C57BL/6 mice by culture in complete RPMI 1640 medium (contains 2 mM glutamine, 50 units/ml penicillin, 50 μg/ml streptomycin, 50 μM 2-mercaptoethanol [all Life Technologies, Renfrew UK] and 10% fetal calf serum [Lonza, Nottingham UK]), supplemented with 10 ng/ml recombinant murine GM-CSF (Peprotech, London UK), at 37 °C in 5% CO_2_. On day 6, immature bmDCs (65–85% CD11c^+^) were harvested by gentle scraping and these bmDCs were further purified (≥95%) by positive selection using CD11c-MACS microbead separation (Miltenyi Biotec, Woking, UK) according to the manufacturer’s instructions. CD11c^+^ DCs (2 × 10^6^ cells) were then incubated in medium ± ES-62 (2 μg/ml) and/or lipopolysaccharide (LPS; 1 μg/ml) (*Salmonella minnesota* or, where indicated, *Escherichia coli*, strain 055:B5 [Sigma, Poole UK]) prior to detection of cytokine release by ELISA and/or cell signalling events by Western Blotting^7^,^8^,^17^,^22^,^57^,^58^.

For bmDC-T cell co-cultures, DCs were incubated in medium ± ES-62 and/or LPS, prior to pulsing with OVA_323–339_ peptide (0–300 nM) before incubation with naive OVA-specific CD4^+^CD62L^+^ T cells from DO.11.10.BALB/c or OT-II.C57BL/6 mice, isolated using Miltenyi magnetic bead technology^4^,^21^,^54^,^55^,^58^. In some experiments, the transgenic (tg) TCR T cells, detected using the clonotypic monoclonal Ab KJ1.26, were labelled with 5, 6-Carboxy-Succinimidyl-Fluorescein-Ester (CFSE; 5 μM) and assessed for cell phenotype and proliferation by flow cytometry as previously described, using a Becton Dickinson FACSCalibar^TM^ flow cytometer (BD Biosciences, Oxford UK) and analysed using Flowjo software (Tree Star Inc, OR, USA, version 8.8.6)^4^,^5^,^21^,^54^.

Where indicated, to block autophagic flux^28^, DCs were treated with a combination of E-64d plus pepstatin A (both 10 μg/ml; Enzo Life Sciences, Exeter UK) to raise the pH of lytic granules and inhibit lysosomal proteases, respectively, NH_4_Cl to neutralise lysosomal pH (50 μM) or 3MA (2 mM) to inhibit the Class III PtdIns-3-K signalling essential for the early stages of autophagy^28^. Alternatively, the proteasomal degradation inhibitor lactacystin (10 μM; Enzo Life Sciences, Exeter UK), which inhibits the ATP independent activities of the proteosome, was also utilised in these studies as we described previously^17^. For the siRNA studies, Qiagen PKC-δ and Allstar-3 control siRNA were used at a final concentration of 1 nM according to Qiagen and PolyPlus manufacturers’ protocols (Qiagen, Manchester UK). Briefly, DCs were plated at 10^4^ cells/well and allowed to rest before being exposed to two treatments with siRNA in INTERFERin siRNA transfection reagent (PolyPLus) at 24 and 48 h and then after a further 2 h, cells were treated with LPS and 18 h later cell supernatants harvested for analysis of cytokine release by ELISA. Cells were also analysed by FACE assays as we described previously^8^ using a rabbit anti-PKCδ (clone 2058; Cell Signalling, Leiden, Netherlands) and a HRP-conjugated anti-rabbit IgG polyclonal antibody (Cell Signalling, Leiden, Netherlands).

For the adoptive transfer studies, bmDCs were prepared as described above and harvested on day 6. Cells (2 × 10^6^/well) were then cultured in 6 well plates in the presence or absence of 2 μg/ml ES-62 and 10 μg/ml (5 μM) OVA_323–339_ peptide. Where indicated, after 24 h, the DCs were further stimulated with 1 μg/ml *E. coli* 055:B5 LPS for 24 h. On day 8, non-irradiated, age matched, male BALB/c recipient mice that had been adoptively transferred with 2.5 × 10^6^ KJ1.26^+^CD4^+^ DO.11.10 T cells i.v.^21^,^54^ one day earlier were then injected s.c. in the left hind footpad with 2.5 × 10^5^ bmDCs.

### ELISA

IL-6, TNF-α (both eBioscience, Hatfield UK), IL-12p40, IL-12p70, IL-22, IL-23 (all R&D, Abingdon UK) and IL-17A (Biolegend, London UK) cytokine ELISA analysis was performed according to the manufacturer’s instructions.

### Western Blotting

Western Blot analysis was carried out as described previously^8^,^17^,^22^,^58^. Briefly, bmDC (2 × 10^6^ cells/treatment) reactions were terminated by washing with ice cold PBS before lysing and solubilising cells in 50 μl modified RIPA buffer (50 mM Tris buffer, pH 7.4 containing 150 mM sodium chloride, 2% (v/v) NP-40, 0.25% (w/v) sodium deoxycholate, 1 mM EGTA, 0.5 mM phenylmethylsulfonylfluoride [PMSF], 1 in 100 dilutions of Halt^TM^ Protease and Phosphatase Inhibitor Cocktails [Thermo Scientific, Renfrew, UK]). Protein concentrations were assessed by the BCA protein assay (Pierce [Thermo Fisher] Renfrew, UK) and equal amounts of samples were resolved and proteins transferred to nitrocellulose membranes using the XCell *SureLock* Mini-cell kit with NuPAGE Novex “high performance” pre-cast Bis-Tris gels and NuPAGE buffers and reagents (all supplied by Invitrogen, Renfrew, UK). Nitrocellulose membranes were blocked in TBS/Tween 20 containing 5% non-fat milk protein and all antibodies were diluted in TBS/Tween 20 with 5% BSA. Protein bands were visualised using the NOVEX^®^ ECL HRP Chemiluminescent detection system (Invitrogen, Renfrew UK) and autoradiography film, with densitometry performed using Image J software. Antibodies with the following specificities were used: p62 (SQSTM1): #NPB1-42821 (Novus Biologicals, Abingdon UK); PKCα: #610107, (BD Bioscience, Oxford UK); PKCδ: #2058; PKCε: #2683; LC3A/B #4018 and HRP-conjugated anti- mouse or anti-rabbit IgG polyclonal antibodies (all Cell Signalling Technologies, Leiden Netherlands). The data presented in the main text figures are cropped blots but representative full-length images of the relevant antibody specificities are shown and cross-referenced in Supplementary Figure 1.

### RT-PCR

Preparation of RNA and consequent RT-PCR analysis was performed as described previously^4^,^32^. Briefly, RNA was extracted using the RNeasy micro kit (Qiaqen, Manchester UK) prior to DNase (Invitrogen, Renfrew UK) digestion and cDNA preparation using the “High Capacity cDNA Reverse Transcription Kit” (Applied Biosystems, ThermoFisher, Renfrew, UK) as per the manufacturer’s instructions. TaqMan RT-PCR was performed in a 7900 HT Sequence Detector (Applied Biosystems, ThermoFisher, Renfrew, UK) using pre-designed Applied Biosystems primer/probe kits (GAPDH, #-4352339E; HPRT, #-4331182; IL-17 Mm00439618_m1 and IL-22 Mm00444241_m1) with thermal cycler conditions as follows: 20 seconds at 95 °C, followed by 45 two-temperature cycles (3 seconds at 95 °C and 30 seconds at 60 °C) with a final step of 60 °C for 30 seconds. Data analysis was completed using RQ Manager Software (Applied Biosystems, ThermoFisher, Renfrew, UK) and expressed as Rq values where the 2^−ΔΔCt values have been determined relative to the house-keeping reference gene (GAPDH) and experimental control samples, namely the medium-only DCs (None; 0 nM OVA peptide).

### Immunofluorescence

Immunofluorescence was performed as described previously^4^,^5^,^32^. Briefly, cells were fixed with 4% paraformaldehyde (PFA), permeabilised with 0.1% (v/v)Triton-x in PBS and incubated with 0.3 M glycine in PBS to reduce cellular autofluorescence. Blocking of non-specific binding was performed with 1% BSA and 10% normal goat serum (Invitrogen, Renfrew UK), or the appropriate species for the secondary antibody, for 30 min and specificity of reactivity validated by the relevant epitope blocking peptide. Cells were counterstained with 4′,6- DAPI and mounted in Pro-long Gold anti-fade mounting medium (Invitrogen, ThermoFisher, Renfrew, UK). Images were acquired and analysed on a Zeiss LSM510 confocal microscope using LSM Image Browser software.

### Statistical Analysis

Parametric data were analyzed by Student’s unpaired t-test or by one-way ANOVA followed by the Bonferroni’s post-test. Normalized data were analyzed by Kruskal-Wallis test.

## Additional Information

**How to cite this article**: Eason, R. J. *et al*. The helminth product, ES-62 modulates dendritic cell responses by inducing the selective autophagolysosmal degradation of TLR-transducers, as exemplified by PKCδ. *Sci. Rep*. **6**, 37276; doi: 10.1038/srep37276 (2016).

**Publisher’s note**: Springer Nature remains neutral with regard to jurisdictional claims in published maps and institutional affiliations.

## Supplementary Material

Supplementary Information
