# Supplementary material for: The helminth product, ES-62 modulates dendritic cell responses by inducing the selective autophagolysosomal degradation of TLR-transducers, as exemplified by PKCδ
Source: Sci Rep. 2016 Nov 21;6:37276. doi: 10.1038/srep37276 (PMC5116678; doi:10.1038/srep37276)
Supplement: Supplementary Information [file srep37276-s1.pdf]

The helminth product, ES-62 modulates dendritic cell responses by inducing the selective autophagolysosomal degradation of TLR-transducers, as exemplified by PKCδ

Russell J Eason[1], Kara S Bell[2], Fraser A Marshall[1], David T Rodgers[1], Miguel A Pineda[1], Christina N Steiger[1], Lamyaa Al-Riyami[2], William Harnett[2*] and Margaret M Harnett[1*]

[1]Institute of Infection, Immunity and Inflammation, University of Glasgow, Glasgow G12 8TA, UK and [2]Strathclyde Institute of Pharmacy and Biomedical Sciences, University of Strathclyde, Glasgow G4 0RE, UK

***Corresponding authors**: Margaret M Harnett, Institute of Infection, Immunity and Inflammation, University of Glasgow, Glasgow G12 8TA; tel: +44 141 330 8413; e-mail: Margaret.Harnett@glasgow.ac.uk

William Harnett, Strathclyde Institute of Pharmacy and Biomedical Sciences, University of Strathclyde, Glasgow G4 0RE; tel: +44 141 548 3725; e-mail: W.Harnett@strath.ac.uk

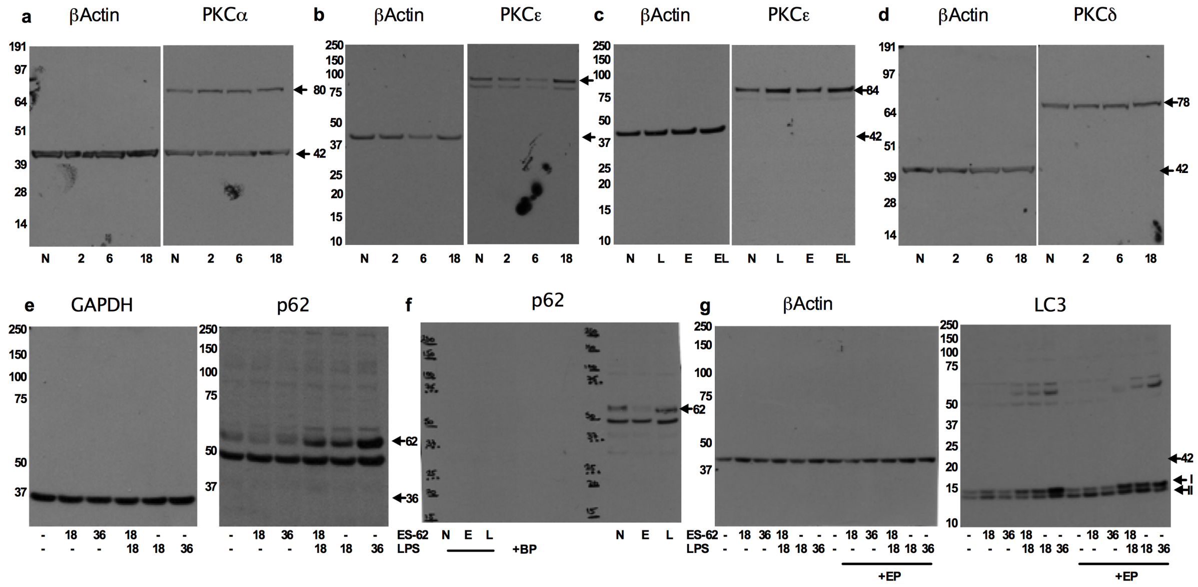

Supplementary Figure 1 Representative full-length images of the Western Blot analysis of expression of PKC-α (a), PKC-ε (**b**) and PKC-δ (**d**) and their βactin loading controls are shown with molecular marker standards. Arrows indicate the protein of interest and its predicted molecular weight. In (**a**) the residual staining of βactin (p42) following stripping and reprobing with anti-PKC-α is evident. The data shown represent the LPS time-courses (2, 6 and 18 h) presented in Figure 3ai, bi and δi. An additional PKC-ε blot (**c**) in which cells were incubated with medium alone (N), ES-62 (E), LPS (L) for 18 h or with ES-62 followed by LPS (EL) and the samples analysed at 36h is shown to highlight that the upper band detected is PKCε, and these data contributed to the statistical analysis in the Figure 3 legend. Full-length versions of the GAPDH and p62 expression presented in Figure 4 are shown in (**e**). The p47 band in the p62 blot is the SQSTM1 domain-containing protein that is recognised by most commercially available antibodies as its expression is also blocked by the p62 blocking peptide (BP; **f**). The LC3-I, LC3-II and βactin data (**g**) are the uncropped images of the experiment presented in Figure 5ai.
